# High Incidence of Burst Suppression during Propofol Sedation for Outpatient Colonoscopy: Lessons Learned from Neuromonitoring

**DOI:** 10.1155/2020/7246570

**Published:** 2020-06-19

**Authors:** Jamie Bloom, David Wyler, Marc C. Torjman, Tuan Trinh, Lucy Li, Amy Mehta, Evan Fitchett, David Kastenberg, Michael Mahla, Victor Romo

**Affiliations:** ^1^Department of Anesthesiology, Division of Neurological Anesthesia, Thomas Jefferson University, 111 S. 11^th^ Street, Philadelphia, PA 19107, USA; ^2^Sidney Kimmel Medical College, 1025 Walnut Street, Philadelphia, PA 19107, USA; ^3^Department of Medicine, Division of Gastroenterology and Hepatology, Thomas Jefferson University, 132 S. 10^th^ Street, Philadelphia, PA 19107, USA

## Abstract

**Background:**

Although anesthesia providers may plan for moderate sedation, the depth of sedation is rarely quantified. Using processed electroencephalography (EEG) to assess the depth of sedation, this study investigates the incidence of general anesthesia with variable burst suppression in patients receiving propofol for outpatient colonoscopy. The lessons learned from neuromonitoring can then be used to guide institutional best sedation practice.

**Methods:**

This was a prospective observational study of 119 outpatients undergoing colonoscopy at Thomas Jefferson University Hospital (TJUH). Propofol was administered by CRNAs under anesthesiologists' supervision. The Patient State Index (PSi™) generated by the Masimo SedLine® Brain Root Function monitor (Masimo Corp., Irvine, CA) was used to assess the depth of sedation. PSi data correlating to general anesthesia with variable burst suppression were confirmed by neuroelectrophysiologists' interpretation of unprocessed EEG.

**Results:**

PSi values of <50 consistent with general anesthesia were attained in 118/119 (99.1%) patients. Of these patients, 33 (27.7%) attained PSi values <25 consistent with variable burst suppression. The 118 patients that reached PSi <50 spent a significantly greater percentage (53.1% vs. 42%) of their case at PSi levels <50 compared to PSi levels >50 (*p*=0.001). Mean total propofol dose was significantly correlated to patient PSi during periods of PSi <25 (*R*=0.406, *p*=0.021).

**Conclusion:**

Although providers planned for moderate to deep sedation, processed EEG showed patients were under general anesthesia, often with burst suppression. Anesthesiologists and endoscopists may utilize processed EEG to recognize their institutional practice patterns of procedural sedation with propofol and improve upon it.

## 1. Introduction

Multiple guidelines support colonoscopy as an effective tool for colorectal cancer screening [1, 2]. Over the past two decades, expansion of screening has occurred primarily through the increased use of colonoscopy [3]. Coinciding with this trend has been the marked rise in the use of anesthesiology services in colonoscopy practice [4].

Propofol is a popular agent for sedation during colonoscopies. Its primary benefits derive from a favorable pharmacokinetic profile, notably a rapid onset and offset. In comparison to benzodiazepine and narcotic-based techniques, propofol sedation allows for quicker in-room to procedure-start time as well as faster recovery and discharge times [5]. However, propofol has a narrow therapeutic index associated with adverse effects including respiratory depression, hypotension, and aspiration related to loss of airway reflexes [4, 6]. A review of the American Society of Anesthesiologists (ASA) Closed Claims database for complications of anesthesia at remote locations showed that sedation leading to respiratory depression comprised over half of gastrointestinal suite claims [7]. Propofol was used in the majority (78%) of those claims.

The 2018 ASA sedation guidelines define moderate sedation as the drug-induced depression of consciousness during which patients respond purposefully to verbal or tactile stimulation. Spontaneous ventilation and cardiovascular function are maintained. In contrast, deep sedation and general anesthesia are states where spontaneous ventilation may be inadequate and cardiovascular function may be impaired [8]. During sedation, it is not always possible to predict how a specific patient will respond to sedative and analgesic medications. With emphasis on patient satisfaction and endoscopic performance, the spectrum of sedation actually attained in gastrointestinal endoscopy procedures may extend to levels deeper than planned for, thereby exposing patients to the aforementioned cardiorespiratory risks [8].

Processed electroencephalogram (EEG) monitors are clinical tools available to anesthesiologists that may offer a more accurate determination of sedation depth than the traditional approach of titrating dose based on patient comfort or movement alone [9]. Specifically, titration of propofol using processed EEG has the potential to optimize endoscopic sedation by lessening time in states of general anesthesia or burst suppression while maintaining the benefits of a propofol-based technique [10, 11]. Processed electroencephalography is a validated modality for the objective assessment of the depth of sedation/unconsciousness. Using frontal EEG recordings and a proprietary algorithm, a processed EEG monitor generates single categorical variables corresponding to different states of anesthesia including consciousness, general anesthesia, and general anesthesia with burst suppression [10, 12]. Burst suppression is the electroencephalographic finding of alternating periods of isoelectricity (suppression) and relatively higher voltage bursts. It is associated with several pathological brain states, as well as general anesthesia or comatose states. As such, burst suppression is a marker for profound brain inactivity [13].

Using processed EEG, this study investigates not only the depth of unconsciousness but also the incidence of general anesthesia with variable burst suppression in patients receiving propofol for outpatient colonoscopy. The aim of this prospective observational study was to use processed EEG to establish the depth of sedation profile attained with propofol in patients undergoing outpatient colonoscopy.

## 2. Materials and Methods

The study protocol was approved by the Institutional Review Board (IRB) at TJUH. A waiver for written consent was approved by the IRB and all participating patients verbally agreed to undergo this monitoring. The study participants were outpatients undergoing elective colonoscopy from April 2017 to October 2018 at a university hospital-based outpatient gastrointestinal suite in which multiple gastroenterologists and anesthesiologists provide care. Subjects were approached regarding participation on dates that research personnel (a research assistant and anesthesiologist) not directly involved in sedating the patients were available. Subjects were outpatients aged 18–65, ASA Physical Status I or II as determined by the supervising attending anesthesiologist, and received sedation with only propofol for colonoscopy. The first 33 patients comprised a pilot phase of the study that served as a proof of concept before further enrollment to a total of 119 patients.

**Figure 1:**
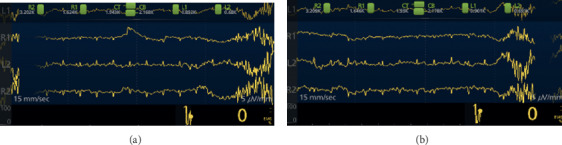
Four lead raw EEG from SedLine® displaying classic 1 : 10 burst to suppression ratio seen in neuroprotection with propofol in two separate patients from this study.

The depth of sedation was monitored with a bihemispheric 4-channel EEG SedLine® Brain Root Function monitor which produces a dimensionless value called the Patient State Index (PSi), an EEG validated measure of the depth of sedation [9]. PSi values can span from 0–100 with higher values indicating a lesser degree of sedation as follows: 0–24 burst suppression with varying degrees of suppression, 25–49 general anesthesia, and ≥50 mild to deep sedation. Validation of PSi values <25 termed as variable burst suppression was confirmed by examination of the raw EEG by a neurointensivist and a neurophysiologist (Figure 1). SedLine® disposable electrodes were placed on the scalp preoperatively after cleaning with 70% isopropanol to limit skin impedance. All subjects and anesthesia personnel in the endoscopy procedure room were blinded to study objectives and PSi data. A member of the study team uninvolved in sedation or the colonoscopy monitored the SedLine® for appropriate operation. Baseline PSi was collected before induction of anesthesia and then monitored continuously during the colonoscopy from start of sedation until completion of sedation as marked by the anesthesia provider in the intraprocedure electronic medical record (EMR). Following the procedure, the raw data from the SedLine® were transferred to an Excel spreadsheet for later analysis.

A propofol-based sedation technique standard to practice at TJUH was followed. At the discretion of the attending anesthesiologist, a certified registered nurse anesthetist (CRNA) administered repeated propofol boluses, a propofol infusion, or a combination of the two methods based on patient age, weight, comorbidities, and practitioner preference with titration to clinical effect. Patients were pretreated with IV lidocaine to prevent propofol-induced phlebitis. No opioids, benzodiazepines, or other sedative agents were administered. Start of sedation was marked with first administration of propofol and conclusion of sedation was marked when the patient responded to verbal commands following completion of the procedure. Time to discharge was defined as the period from end of sedation to time of discharge from the postanesthesia care unit and was recorded. Doses and infusion rates of all medications were recorded in the intraoperative EMR.

Physiologic data including arterial oxygen saturation by pulse oximetry, heart rate, and blood pressure were automatically entered into the EMR. Patients were administered 4–6L O_2_ via nasal cannula and episodes of desaturation were defined as SpO_2_ ≤92%. Any need for airway management including insertion of a nasal airway, chin lift or jaw thrust maneuvers, or airway rescue with placement of a laryngeal mask airway (LMA) or endotracheal tube (ETT) was recorded. Hypotensive events in which mean arterial pressure (MAP) deviated >20% below the preoperative MAP were also recorded.

### 2.1. Data Analysis

The approach to sample size determination was based on the assumption that approximately 35% of case time, in patients having colonoscopy with sedation, would be spent at a level of consciousness defined as general anesthesia (PSi <50) compared to an estimated 65% of case time spent with a PSi ≥50. Those estimates were considered reasonable based on the clinical experience of the investigators and their initial use of the SedLine® monitor. The sample size was determined using a power analysis with a test for equality of two proportions with alpha set at 0.05 and a power of 0.80. With 43 subjects per group (*N* = 86), this study had 80% power to detect a difference in patients assessed as having achieved a state of general anesthesia versus sedation (PSi >50) using the Masimo SedLine® Brain Function monitor.

Descriptive statistics for 119 patients are presented in tables using means ± SD and 95% confidence intervals (CI) of the mean. Demographic data were analyzed using one-way ANOVA and the Kruskal–Wallis test to examine effect of sex. PSi data were analyzed using one-way ANOVA to determine differences between PSi levels and percent of case duration between levels. The *p* value was set at <0.05 for statistical significance and all tests were two-sided. Statistical analyses were performed using SYSTAT version 13 and GraphPad Prism version 6.0.

## 3. Results

**Figure 2:**
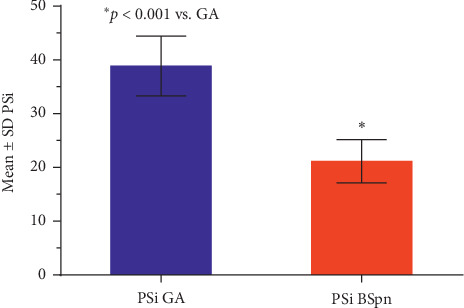
Patients' means ± SD PSi during BSpn (PSi <25) were significantly (*p* < 0.001) lower than their GA (PSi 25–50) for the case.

Demographic data for the study group are presented in Table 1. The study sample consisted of 47 males and 72 females with a mean age of 52.1 years and BMI of 28.9, respectively. The median American Society of Anesthesiologists (ASA) Physical Status was 2.0 with no statistically significant (*p*=0.415) differences between males and females. Similarly, age and BMI were not significantly different between males and females (*p*=0.581 and *p*=0.350). PSi values of <50, consistent with general anesthesia, were attained in 118 of 119 (99.1%) patients. Of these 118 patients, 33 (27.7%) attained PSi values <25 consistent with variable burst suppression (Table 2). The mean PSi reached during these periods of PSi <25 was significantly lower than the mean PSi during periods of PSi 25–50 (21.1 vs. 38.8, *p* < 0.001) (Figure 2).

**Figure 3:**
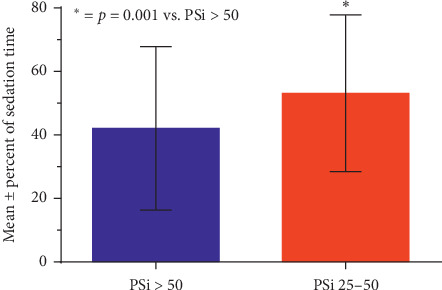
Mean ± SD percent of total case time spent with PSi 25–50 compared to percent of case time at PSi ≥50. The percent of time patients had PSi 25–50 was significantly higher than the percent of time PSi was >50.

**Figure 4:**
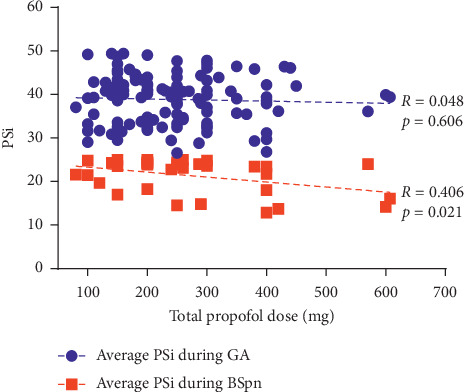
The PSi measures are shown for the episodes of GA and BSpn versus total propofol dose. Propofol dose was not related to the patients' PSi during GA periods (*R* = 0.048, *p*=0.606); however, it was significantly correlated to the patients' PSi during BSpn (*R* = 0.406, *p*=0.021).

The 118 patients that reached PSi <50 spent a significantly greater percentage of their case duration at PSi levels <50 compared to PSi levels ≥50 targeted for moderate to deep sedation (53.1% vs. 42.0%, *p* < 0.001) (Figure 3). Mean total propofol dose administered was 3.1 mg/kg for these relatively short cases. The procedures were generally completed within 30 minutes with little time variability between cases demonstrated by a standard deviation of 6.7 minutes. The total propofol dose was not correlated to patient PSi during periods of PSi 25–49 but was significantly correlated to patient PSi during periods of PSi <25 (*R* = 0.406, *p*=0.021) (Figure 4).

Significant hypotension with greater than a 20% decrease in MAP from baseline was observed in 62 patients (52%) during the course of their sedation. In these patients, baseline mean MAP differed significantly both from overall mean intraoperative MAP and mean MAP during periods of hypotension. Mean PSi during periods of PSi 25–49 was not correlated to MAP during episodes of hypotension. There were 13 instances of hypoxemia necessitating rescue with either insertion of a nasal airway or performance of chin lift maneuver to restore normoxemia. There were no instances that required airway rescue with a LMA or ETT. Mean case duration was 17.5 min and mean time to discharge was 54.7 min. Mean PSi during periods of PSi 25–49 was not correlated to time to discharge.

## 4. Discussion

Utilizing processed EEG (PSi from SedLine®), this study determined that patients undergoing colonoscopy with propofol sedation reach levels of sedation that are deeper than guidelines suggest for colonoscopy. Only one subject in this 119 patient study did not enter general anesthesia or burst suppression during their colonoscopy. Subsequent investigation revealed that the CRNA administering propofol for this unique case was primarily using an infusion and did not administer boluses of propofol after starting the infusion. This limited, anecdotal information may suggest that, with refined technique, conscious sedation using propofol is an achievable goal. However, 118 subjects (99.1%) reached a level of sedation consistent with general anesthesia, and over twenty-five percent experienced a level seen with variable burst suppression. Feedback such as the aforementioned maybe utilized to optimize institutional sedation practice.

Our findings underscore the assertion that level of sedation for most colonoscopy procedures exceeds moderate to deep sedation. In fact, an average of only 42.0% of case duration was spent at the targeted depth of moderate to deep sedation. Similarly, in a 100 patient double-blinded prospective study by Ramsey et al., in which depth of sedation during propofol-based GI sedation was assessed by PSi and the Ramsey sedation score (RSS), a subjective sedation scale, 89% of subjects were under general anesthesia accounting for 47% of total procedure time [14].

Given the depth of sedation achieved in our study, anesthesia providers should be aware that the depth of sedation achieved using propofol for common endoscopic procedures may be deeper than anticipated. Accordingly, when anesthesia providers administer propofol, ASA practice guidelines advise that care be consistent with that required for general anesthesia when using agents intended for general anesthesia [8]. This would entail the presence of individuals with expertise in advanced airway management and trained to recognize and treat cardiopulmonary complications. In addition, given the results of this study, one could argue that the preoperative consent process should include a discussion of general anesthesia and its complications. In the study reported herein, slightly over half the subjects experienced *a* >20% decrease in MAP from their baseline. Despite administration of oxygen via nasal cannula, approximately 10% of patients experienced desaturation below 92% SpO_2_ necessitating brief airway maneuvers to restore oxygenation.

Although the abovementioned concerns may seem alarming, the clinical significance of deeper than intended propofol sedation in GI endoscopy procedures remains unclear. In this study, there were no obvious major negative clinical sequelae suffered by any patients despite episodes of hypotension and desaturation and attainment of general anesthetic levels of sedation. This was a small study underpowered for the detection of complications, but other large studies of nonanesthesia administered propofol sedation have shown that propofol-related complications associated with routine endoscopy remain rare. In one retrospective study of ∼36,000 patients, no patient required intubation or died and <0.2% required assisted ventilation [11]. In a prospective study of 24,000 patients, a major adverse event rate of 0.016% was observed [15].

Nonetheless, when propofol sedation complications do occur, this study offers potential insight towards an etiology. For instance, while there was no evidence for aspiration in this study, the rare complication of aspiration pneumonia following colonoscopy has been found to be high with anesthesia assistance [4, 16]. The unintended extensive depth and breadth of sedation reached in our study's patients may at least partially explain this phenomenon.

There were limitations of this study. Most notably, there was no process that controlled for colonoscopy sedation based on indication (screening versus symptom initiated), colonoscopist skill, technically difficult procedures, length of procedures, stimulating events during procedures such as insertion, or home medications responsible for drug-drug interactions with anesthetics. Each of these factors could impact the amount of sedation administered to subjects. Future research should also include a multicenter study to eliminate provider practice bias and geographical practice bias. Furthermore, we examined the depth of anesthesia using solely propofol. Since propofol lacks analgesic effects, larger doses and thus deeper sedation may be required in the absence of opioids that typically enhance the anesthetic regimen [11]. This single-drug approach may not be representative of anesthetic techniques employed for endoscopy by other institutions and may also represent a deviation from our own practitioners' standard practices for conscious sedation outside the endoscopy setting.

The effect of blinding anesthesia personnel to PSi and study objectives was not assessed. The presence of an investigator could bias an anesthetic provider to giving a larger or smaller dose to conform to a perceived study benefit. A future study could address this possible concern for bias by surveying anesthetic providers with a questionnaire to elicit their response to investigator presence on propofol dose. Interestingly, in this study, the total propofol dose was significantly correlated to mean PSi during periods of PSi <25. Had providers become unblinded (and aware of patient PSi changes), intraprocedural adjustments to propofol dosing and depth of sedation could have been made. Further research to elucidate the impact of PSi monitoring on anesthetist practice would clarify whether the data provided by the Masimo SedLine could have clinical utility either for avoiding overly deep anesthesia or avoiding unnecessary propofol redosing when favorable clinical conditions are being met.

Given the results of this study, it is the opinion of the authors that feedback from neuromonitoring along with information obtained from the clinical examination can help target best sedation practice at an institution. One systematic review by Shephard et al. demonstrated that while anesthetic depth monitors appear to offer improvement in cost-effectiveness by reducing consumption of anesthetic agents and shortening anesthetic time, their benefit in warning of adverse events and reducing intraoperative awareness is limited [17]. New ASA guidelines for moderate procedural sedation include recommendations for monitoring patient level of consciousness that rely on response to verbal or tactile stimulation for indication of consciousness. These guidelines did not include a literature review on the depth of anesthesia monitors as part of the portion dedicated to monitoring level of consciousness.

## 5. Conclusions

The depth of sedation achieved with anesthesia administered propofol for colonoscopy spans a continuum. Although providers planned for moderate to deep sedation, processed EEG in this study revealed a substantially greater depth consistent with general anesthesia and even burst suppression. Anesthesia professionals and endoscopists should recognize that propofol administration to achieve favorable procedural conditions may require classification as general anesthesia and therefore should consider consenting patients for general anesthesia. Feedback from devices such as employed in this study to guide the depth of sedation may be considered to not only raise awareness of the physiologic implications of deeper levels of sedation but also to optimize institutional sedation practice. Further research is required to establish the impact on patient outcomes of anesthetic practices resulting in intraprocedural burst suppression.

## Figures and Tables

**Table 1:** Descriptive statistics for demographic and dosing variables for the study group.

	*N*	Range	Mean	Standard deviation	95.0% CI of mean
*Demographics*					
Male/female	47/72	—	—	—	—
Age	118	25.0–65.0	52.1	9.2	50.4–53.7
ASA status	118	1-2	1.8	0.3	1.7–1.9
BMI	119	19.0–67.4	28.9	6.3	27.7–30.0
Height (m)	119	1.4–2.0	1.6	0.1	1.6-1.7
Weight (kg)	119	49.0–201.0	83.3	20.3	79.6–87.0

*Dosing*					
Number of propofol boluses	86	1.0–8.0	2.5	1.2	2.2–2.7
Total propofol dose (mg)	118	80.0–607.2	242.9	105.7	223.6–262.1
Propofol dose per kg (mg)	119	0.7–9.3	3.1	1.5	2.8–3.4
Surgical duration (min)	119	7.0–46.4	17.5	6.7	16.3–18.7

**Table 2:** Descriptive statistics for PSi data.

	*N*	Range	Mean	Standard deviation	95.0% CI of mean	*p* value
Mean PSi during GA: PSi 25–50	118	26.5–49.4	^*∗*^38.8	5.5	37.8–39.9	^*∗*^<0.001
Mean PSi during deep GA: PSi <25	33	12.8–25.0	^*∗*^21.1	4.0	19.7–22.6	

Percent of case in sedation: PSi >50	118	2.6–100.0	^+^42.0	25.7	37.3–46.7	^+^<0.001
Percent of case during GA: PSi 25–50	118	2.0–97.3	^+^53.1	24.6	48.6–57.6	
Percent of case during deep GA: PSi <25	33	0.4–77.5	^+^20.6	21.2	13.1–28.1	

^*∗*^Statistical significance between the two means. ^+^The three means to be significantly different from one another.

## Data Availability

All data can be requested from the Thomas Jefferson University Department of Anesthesiology.

## Abbreviations

EEG: Electroencephalography
MAC: Monitored anesthesia care
PSi: Patient State Index
CRNA: Certified registered nurse anesthetist
ASA: American Society of Anesthesiologists
TJUH: Thomas Jefferson University Hospital
IRB: Institutional Review Board.

## Additional Points

*Key Points*. Question: what is the incidence of general anesthesia and variable burst suppression in patients receiving propofol for sedation during outpatient colonoscopies? Findings: general anesthesia occurred in 118/119 (99.1%) cases; of these, 33 (27.7%) cases were characterized by EEG findings consistent with variable burst suppression. Meaning: anesthesia professionals and endoscopists should recognize that propofol administration to achieve favorable procedural conditions may produce intermittent states of overly deep general anesthesia characterized by variable burst suppression on EEG. In the setting of endoscopy, this may inadvertently result in patients' inability to maintain normal minute ventilation or adequately protect their airways.

## References

[1] Rex D. K., Johnson D. A., Anderson J. C., Schoenfeld P. S., Burke C. A., Inadomi J. M. (2009). American college of gastroenterology guidelines for colorectal cancer screening 2008. *American Journal of Gastroenterology*.

[2] Rex D. K., Boland C. R., Dominitz J. A. (2017). Colorectal cancer screening: recommendations for physicians and patients from the U.S. Multi-society task force on colorectal cancer. *Gastroenterology*.

[3] Shahidi N., Cheung W. Y. (2016). Colorectal cancer screening: opportunities to improve uptake, outcomes, and disparities. *World Journal of Gastrointestinal Endoscopy*.

[4] Cooper G. S., Kou T. D., Rex D. K. (2013). Complications following colonoscopy with anesthesia assistance. *JAMA Internal Medicine*.

[5] Ulmer B. J., Hansen J. J., Overley C. A. (2003). Propofol versus midazolam/fentanyl for outpatient colonoscopy: administration by nurses supervised by endoscopists. *Clinical Gastroenterology and Hepatology*.

[6] Amornyotin S. (2013). Sedation and monitoring for gastrointestinal endoscopy. *World Journal of Gastrointestinal Endoscopy*.

[7] Metzner J., Domino K. B. (2010). Risks of anesthesia or sedation outside the operating room: the role of the anesthesia care provider. *Current Opinion in Anaesthesiology*.

[8] American Society of Anesthesiologists (2018). Task force on moderate procedural sedation and analgesia, American association of oral and maxillofacial surgeons, American college of radiology, American dental association, American society of dentist anesthesiologists, and society of interventional radiology, practice guidelines for moderate procedural sedation and analgesia. *Anesthesiology*.

[9] Goudra B., Nuzat A., Singh P. M., Borle A., Carlin A., Gouda G. (2017). Association between type of sedation and the adverse events associated with gastrointestinal endoscopy: an analysis of 5 years’ data from a tertiary center in the USA. *Clinical Endoscopy*.

[10] Goudra B., Singh P. M., Gouda G., Borle A., Carlin A., Yadwad A. (2016). Propofol and non-propofol based sedation for outpatient colonoscopy-prospective comparison of depth of sedation using an EEG based SEDLine monitor. *Journal of Clinical Monitoring and Computing*.

[11] Rex D. K., Heuss L. T., Walker J. A., Qi R. (2005). Trained registered nurses/endoscopy teams can administer propofol safely for endoscopy. *Gastroenterology*.

[12] Drover D., Ortega H. R. (2006). Patient state index. *Best Practice & Research Clinical Anaesthesiology*.

[13] Ching S., Purdon P. L., Vijayan S., Kopell N. J., Brown E. N. (2012). A neurophysiological-metabolic model for burst suppression. *Proceedings of the National Academy of Sciences*.

[14] Ramsay M. A. E., Newman K. B., Jacobson R. M. (2014). Sedation levels during propofol administration for outpatient colonoscopies. *Baylor University Medical Center Proceedings*.

[15] Sieg A., Beck S., Scholl S. G. (2014). Safety analysis of endoscopist-directed propofol sedation: a prospective, national multicenter study of 24 441 patients in German outpatient practices. *Journal of Gastroenterology and Hepatology*.

[16] Early D. S., Lightdale J. R., Vargo J. J. (2018). Guidelines for sedation and anesthesia in GI endoscopy. *Gastrointestinal Endoscopy*.

[17] Shepherd J., Jones J., Frampton G. K., Bryant J., Baxter L., Cooper K. (2013). Clinical effectiveness and cost-effectiveness of depth of anaesthesia monitoring (E-entropy, bispectral index and narcotrend): a systematic review and economic evaluation. *Health Technol Assess*.

